# Broadly reactive antibodies against influenza B virus hemagglutinin neutralize and protect through distinct structural mechanisms

**DOI:** 10.1128/jvi.00802-26

**Published:** 2026-09-04

**Authors:** Disha Bhavsar, Alesandro Civljak, Bruno Bonnettaz, Guha Asthagiri Arunkumar, Florian Krammer, Goran Bajic

**Affiliations:** 1 Department of Microbiology, Icahn School of Medicine at Mount Sinai 5925 , New York, New York, USA; 2 Center for Vaccine Research and Pandemic Preparedness, Icahn School of Medicine at Mount Sinai 5925 , New York, New York, USA; 3 Department of Pathology, Molecular and Cell-Based Medicine, Icahn School of Medicine at Mount Sinai 5925 , New York, New York, USA; 4 Ignaz Semmelweis Institute, Interuniversity Institute for Infection Research, Medical University of Vienna, Vienna, Austria; 5 Ludwig Boltzmann Institute for Science Outreach and Pandemic Preparedness, Medical University of Vienna, Vienna, Austria; University of Minnesota Twin Cities, Minneapolis, Minnesota, USA

**Keywords:** viral glycoprotein, cryo-EM, vaccine, antibody, influenza B

## Abstract

Influenza B viruses contribute substantially to seasonal disease burden; however, the structural basis by which antibodies recognize the major glycoprotein hemagglutinin (HA) and mediate antiviral activity remains incompletely defined. Influenza B virus used to circulate as two antigenically distinct lineages, B/Victoria/2/1987-like and B/Yamagata/16/1988-like, although the latter has not been detected in global surveillance in recent years. Antigenic drift in HA contributes to reduced vaccine effectiveness; however, the structural and functional basis by which antibodies recognize influenza B virus HA and mediate antiviral activity remains incompletely defined and thus thwarts our efforts in guiding next-generation vaccine design for broad protection. We characterize four murine monoclonal antibodies (mAb) that broadly bind and neutralize influenza B viruses spanning isolates across four decades of antigenic drift. Using cryo-electron microscopy coupled with *in vitro* and *in vivo* functional assays, we show that these antibodies target distinct regions of HA and confer antiviral activity through multiple mechanisms. One antibody engages the receptor-binding site and potently inhibits hemagglutination, whereas others interfere with viral egress and inhibit neuraminidase (NA) activity, suggesting steric occlusion of NA. A medial-junction antibody additionally induces antibody-dependent cellular cytotoxicity *in vitro*. Despite these mechanistic differences, all antibodies confer complete protection in mice when administered prophylactically or therapeutically. Together, these findings define distinct modes of antibody recognition of influenza B virus HA and link epitope specificity to antiviral function, providing a mechanistic understanding of correlates of immune protection and informing efforts to elicit broadly protective antibody responses against influenza B viruses.

**IMPORTANCE:**

Influenza B viruses cause substantial seasonal illness, particularly in children; however, antibody responses against influenza B virus remain less well understood than those against influenza A virus. Here, we identified four antibodies that broadly recognize influenza B virus hemagglutinin and protect through distinct mechanisms. We determined cryo-electron microscopy structures of three antibody-hemagglutinin complexes to define their epitopes and explain their molecular mechanisms of action. One antibody blocks viral attachment by engaging the receptor-binding site, whereas antibodies targeting the medial junction act through post-entry antiviral activity, neuraminidase inhibition, or immune effector functions. Although the antibodies differed in neutralizing potency, all protected mice when administered before infection, and several remained effective after infection. These findings show that broad protection against influenza B virus can arise through multiple antibody targets and mechanisms, informing the evaluation and design of future vaccines and antibody-based therapies.

## INTRODUCTION

Influenza A and influenza B viruses co-circulate seasonally and together impose a substantial global burden of morbidity and mortality despite widespread vaccination (1–4). Although influenza A viruses have historically dominated vaccine design efforts, influenza B viruses contribute a significant and sometimes predominant fraction of disease, particularly in pediatric populations (5–7). Influenza B viruses diverged into two antigenically distinct lineages, B/Victoria/2/1987-like and B/Yamagata/16/1988-like, which co-circulated for decades (8) and were both included in seasonal quadrivalent vaccines (9). However, since the onset of the coronavirus disease 2019 (COVID-19) pandemic, B/Yamagata/16/1988-like lineage viruses have not been detected in global surveillance, likely due to low fitness in combination with reduced transmission associated with changes in social behavior (10, 11). Whether this lineage has been completely eliminated from circulation in the human population remains unclear. This apparent contraction of influenza B virus diversity highlights the dynamic and incompletely understood antigenic landscape of these viruses that extends beyond antigenic drift (12, 13).

Influenza virus hemagglutinin (HA), the major surface glycoprotein, mediates receptor binding and membrane fusion and is the primary target of protective antibody responses (14–16). Structural studies of influenza A virus HA have defined conserved antigenic sites and revealed mechanisms by which antibodies achieve breadth and neutralization, including targeting of the receptor-binding site (RBS), lateral patch, trimer interface, and stem epitopes (17–23). These studies have provided a framework for understanding humoral immunity and have guided efforts toward broadly protective vaccine strategies (24–27). In contrast, the structural basis of antibody recognition of influenza B virus HA remains comparatively underdeveloped. Although broadly reactive antibodies targeting both head and stem epitopes have been described (28–30), the structural principles that govern cross-lineage recognition, neutralization, and functional diversity are not well defined.

Influenza B viruses evolve more slowly than influenza A viruses but still generate sufficient antigenic diversity within and across lineages to challenge durable antibody-mediated protection (12, 31, 32). Fundamental questions remain about how antibodies achieve breadth across this antigenic space. In particular, the relationship between epitope specificity, mechanism of action, and protective function for influenza B virus-specific antibodies remains unclear. Defining these relationships is essential for understanding how host humoral immunity to influenza B virus is shaped and how it can be leveraged toward intervention strategies for the development of broader protection.

Here, we define the structural and functional landscape of antibody recognition of influenza B virus HA by characterizing monoclonal antibodies (mAbs) derived from mice exposed to sub-lethal doses of influenza B viruses. The isolated antibodies display an extraordinary binding breadth spanning isolates from 1940 to 2018, target both the head and the stem regions of HA, and exhibit distinct mechanisms of protection against influenza B viruses. We show that a head-directed antibody engages the RBS and directly interferes with receptor attachment, whereas antibodies targeting the medial junction interfere with viral egress and provide protection through effector function. Together, these findings provide insight into how antibodies engage influenza B virus HA and inform efforts to elicit broadly protective antibody responses.

## RESULTS

### Isolation of monoclonal antibodies against influenza B virus hemagglutinin

We previously described 22 broadly cross-reactive, non-neutralizing murine mAbs against influenza B virus HA, most of which targeted the conserved stem domain and protected *in vivo* through Fc-dependent effector activity rather than direct neutralization (33). Here, we focused on the remaining four mAbs with broad reactivity across influenza B virus isolates from 1940 to 2018. These antibodies were generated by hybridoma fusion after sequential sublethal infection of BALB/c mice with B/Lee/1940, B/Malaysia/2506/2004, and B/Yamagata/16/1988 (HA in a 7:1 A/PR/8/1934 H1N1 backbone) viruses and were identified by screening for cross-reactive binding to purified virions and recombinant HAs.

### Four antibodies retain broad binding to influenza B viruses

Binding breadth was assessed by enzyme-linked immunosorbent assay (ELISA) against a panel of purified influenza B viruses spanning the ancestral strain and both antigenic lineages from 1940 to 2018. The degree of HA sequence conservation and phylogenetic relationships among these viruses are shown in Fig. 1A. While all four antibodies displayed a very broad binding breadth against influenza B virus isolates from 1940 to present, KL-BHA-3A7 and KL-BHA-4C2 consistently exhibited stronger binding (lower minimum binding concentrations) across the panel than KL-BHA-3F4 and KL-BHA-3D7 (Fig. 1B). Notably, all four antibodies retained binding to recent clinical isolates, including B/New York/PV01181/2018 (B/Victoria/2/1987-like lineage) and B/New York/PV00094/2017 (B/Yamagata/16/1988-like lineage). The broad reactivity of these mAbs to recent B/Yamagata/16/1988-lineage viruses was particularly striking, given that the corresponding immunogen was the older B/Yamagata/16/1988 HA. In contrast, several antibodies from our previously described panel of 22 broadly reactive mAbs showed markedly reduced binding to the more recent B/Yamagata/16/1988-like lineage strain B/Phuket/3073/2013 (33). To define the general binding region of these mAbs on HA, we performed ELISAs using a recombinant chimeric cH8/B hemagglutinin composed of an exotic H8 head domain from A/mallard/Sweden/24/2002 and the stem domain of influenza B/Yamagata/16/1988 HA (34). Because the H8 head shares minimal conservation with influenza B virus HA, binding to this construct indicates recognition of the influenza B virus HA stem domain. KL-BHA-3A7 and KL-BHA-4C2 bound the chimeric HA, demonstrating that their epitopes are retained within the influenza B virus HA stem component; structural analysis described below places the KL-BHA-4C2 epitope within the medial junction. KL-BHA-3D7 and KL-BHA-3F4, on the other hand, did not show detectable binding (Fig. S1A). Together, these data indicate that the epitopes targeted by KL-BHA-3A7, KL-BHA-3D7, KL-BHA-3F4, and KL-BHA-4C2 remain highly conserved across influenza B virus lineages and over time.

**Fig 1:**
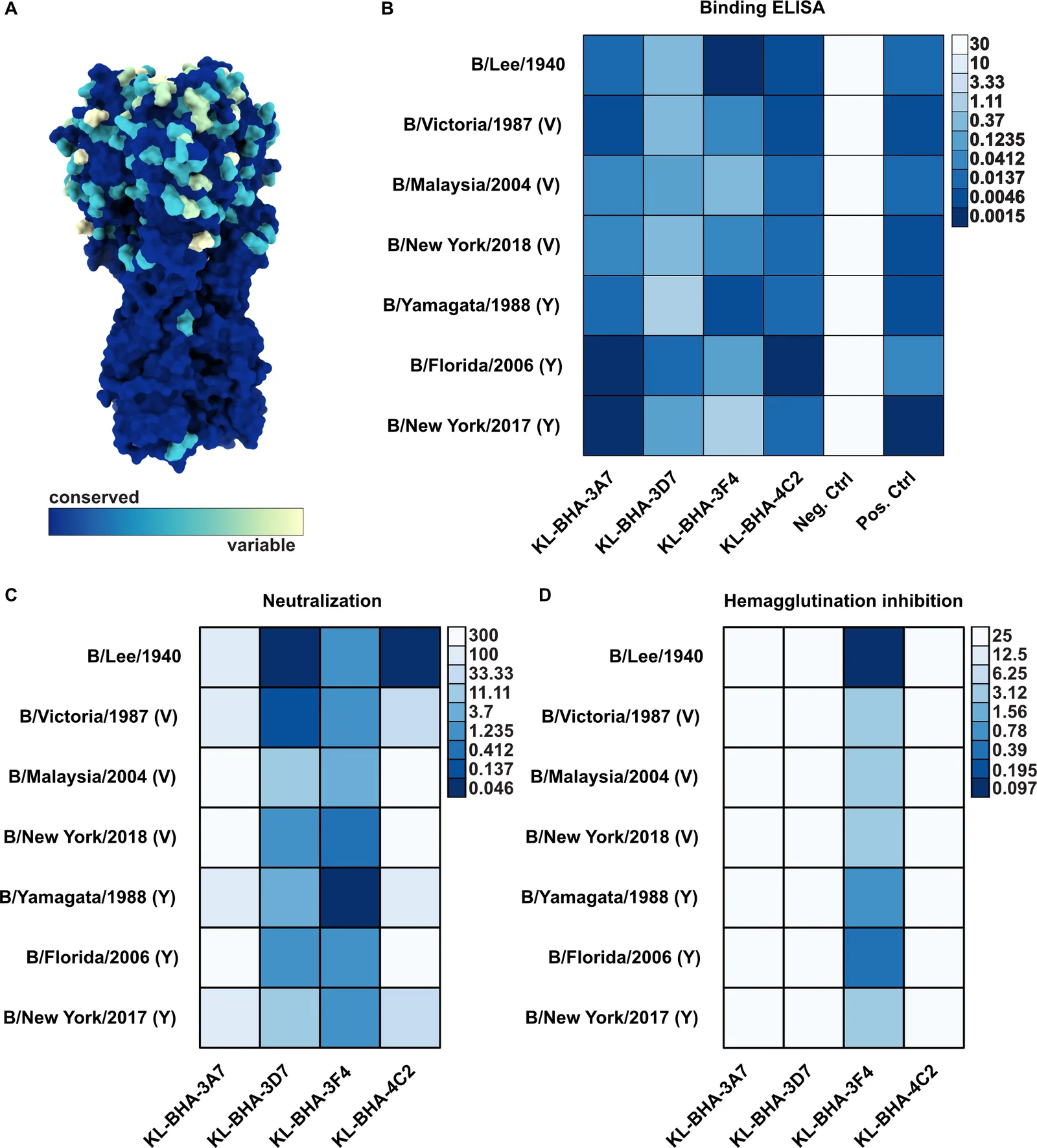
Broad binding and neutralizing activity of anti-influenza B virus HA monoclonal antibodies. (**A**) Amino acid conservation across seven ancestral, B/Victoria/2/1987-like (V), and B/Yamagata/16/1988-like (Y) influenza B virus HAs (1940–2018) mapped onto the B/Brisbane/60/2008 HA structure (PDB ID 4FQM), with least conserved residues in pale yellow and fully conserved residues in dark blue. (**B**) Binding of four anti-influenza B virus HA mAbs to the panel of viruses assessed by ELISA against purified virions. Minimum binding concentrations (µg/mL) are shown. An anti-influenza B virus neuraminidase (NA) mAb (KL-BNA-4F11) and an anti-H6 mAb (8H9) served as positive and negative controls, respectively. (**C**) Neutralization of influenza B viruses by the four mAbs measured by plaque reduction assays across the same panel of strains. IC₅₀ values (µg/mL) derived from non-linear regression demonstrate broad antiviral activity across lineages. (**D**) Hemagglutination inhibition (HAI) activity of the mAbs against the tested viruses. Minimum inhibitory concentrations (µg/mL) are shown, with 25 µg/mL indicating no detectable activity. Strain names in panels B, C, and D are abbreviated; full strain names can be found in Materials and Methods.

### Neutralizing activity of four broadly reactive influenza B virus HA monoclonal antibodies identifies a receptor-blocking antibody

To determine whether the mAbs directly neutralize influenza B viruses, we performed plaque reduction assays. Neutralizing activity was quantified as the reduction in plaque number relative to that observed with an irrelevant control IgG (Fig. 1C). All four mAbs exhibited detectable neutralizing activity at the highest concentration tested (100 µg/mL). KL-BHA-3A7 and KL-BHA-4C2 produced more modest effects overall, with ~30%–50% reductions in plaque number at this concentration, although KL-BHA-4C2 potently neutralized B/Lee/1940 across multiple concentrations. In contrast, KL-BHA-3F4 and KL-BHA-3D7 more consistently suppressed viral replication at lower concentrations. KL-BHA-3D7 also appeared to neutralize B/Victoria/2/1987-like lineage viruses more effectively than B/Yamagata/16/1988-like lineage viruses, with reduced activity against the recent B/New York/PV00094/2017 isolate. Analysis of half-maximal inhibitory concentration (IC_50_) values was consistent with these trends (Fig. S1). For KL-BHA-3A7 and KL-BHA-4C2, IC_50_ values generally ranged from ~50 to 200 µg/mL across the virus panel, with the notable exception of KL-BHA-4C2 against B/Lee/1940. To assess whether neutralization involved inhibition of HA receptor engagement, we performed hemagglutination inhibition (HAI) assays, which measure interference with HA-sialic acid interactions at the receptor binding site (RBS). Among the four antibodies, KL-BHA-3F4 was the only mAb with detectable HAI activity *in vitro*. This activity was observed against both the ancestral B/Lee/1940 strain and lineage-representative viruses, indicating recognition of a conserved functional site. KL-BHA-3F4 showed its strongest HAI activity against B/Lee/1940, consistent with its strong apparent binding in the whole-virion ELISA. Similarly, its robust HAI activity against B/Florida/04/2006 paralleled potent neutralization in plaque reduction assays. However, these relationships were not uniform across all viruses or antibodies, indicating that apparent binding strength and HAI activity do not fully predict neutralization. Together, these data show that KL-BHA-3F4 and KL-BHA-3D7 neutralize viruses from the ancestral, B/Victoria/2/1987-like, and B/Yamagata/16/1988-like groups, whereas KL-BHA-3A7 exhibits only modest neutralizing activity, and KL-BHA-4C2 neutralizes the ancestral B/Lee/1940 strain more effectively than the lineage-representative viruses.

### The mAbs mediate antiviral activity through multiple mechanisms

KL-BHA-3D7 lacked detectable hemagglutination inhibition activity, indicating that its epitope lies outside the RBS of HA. Notably, viruses propagating in the presence of KL-BHA-3D7 formed smaller plaques, suggesting an effect on a post-entry step of the viral life cycle. We postulated that KL-BHA-3D7 restricts viral egress rather than receptor engagement. To test this, we used microneutralization assays designed to distinguish inhibition of viral entry from inhibition of viral egress. Antibodies were added either before infection of Madin-Darby tcanine kidney (MDCK) cells with B/Lee/1940 to assess entry inhibition or after infection to assess effects on viral egress. Neutralization was measured by hemagglutination-based readout. KL-BHA-3D7 neutralized virus in the combined entry-and-egress setting, with activity mapping predominantly to the egress phase (Fig. 2A). Consistent with its lack of HI activity, KL-BHA-3D7 did not inhibit viral entry. Unexpectedly, KL-BHA-3F4 also retained activity when added after the initial infection. Because this assay does not distinguish inhibition of virion release from blockade of progeny-virus attachment, this phenotype may reflect inhibition of viral re-entry and onward spread, consistent with its HAI activity. Its moderate neuraminidase-inhibitory activity may also contribute partially to reduced virion release. The low neutralizing potency of KL-BHA-3A7 and KL-BHA-4C2, even in the plaque reduction assay described above, likely precluded these mAbs from showing activity in the micronuetralization assay described here.

**Fig 2:**
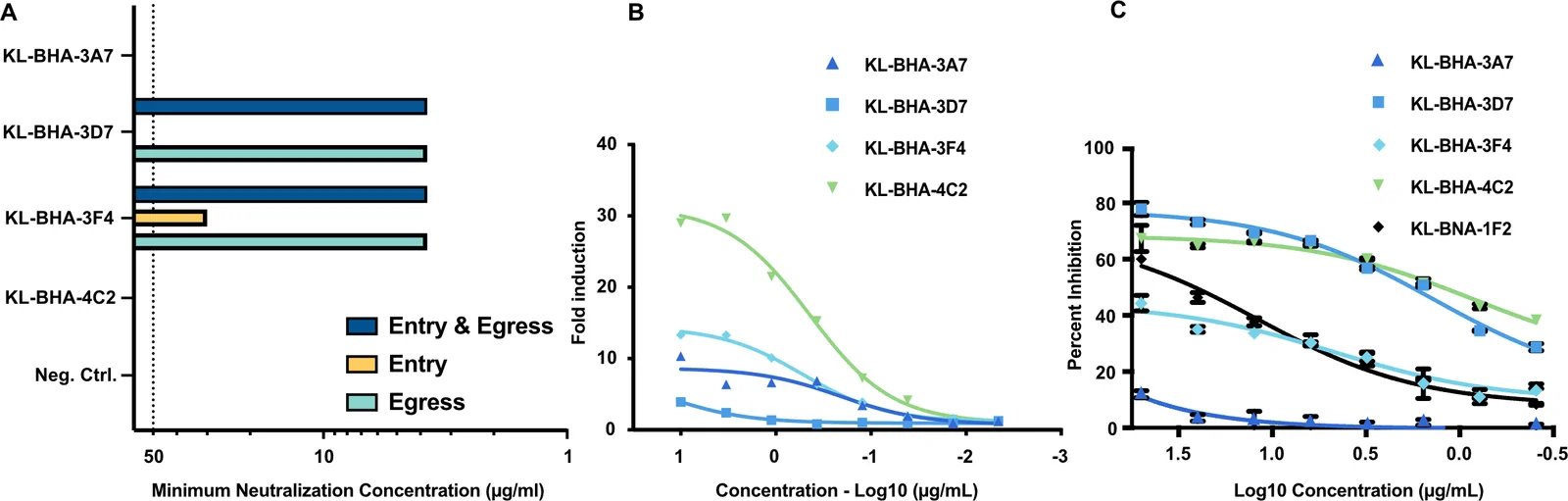
Anti-influenza B virus HA monoclonal antibodies mediate antiviral activity through multiple mechanisms. (**A**) Inhibition of viral entry and/or egress assessed by microneutralization assays. Minimum neutralizing concentrations (µg/mL) were determined using a hemagglutination-based readout. An anti-H6 mAb (8H9) served as a negative control. (**B**) Induction of antibody-dependent cellular cytotoxicity (ADCC) measured using a reporter assay. MDCK cells infected with influenza B/Malaysia/2506/2004 (B/Victoria/2/87-like lineage) were incubated with mAbs, and activity is shown as fold induction over the anti-H6 control (8H9) based on a luciferase readout. (**C**) Neuraminidase inhibition by the mAbs measured against influenza B/Lee/1940 virus. Percent inhibition was normalized to virus-only (0%) and no-virus (100%) controls, with an anti–influenza B virus neuraminidase mAb (KL-BNA-1F2) as a positive control.

We next assessed whether the two cH8/B-reactive antibodies, KL-BHA-3A7 and KL-BHA-4C2, could engage Fc-dependent effector functions by measuring antibody-dependent cellular cytotoxicity (ADCC) activity *in vitro* (Fig. 2B). Using an ADCC reporter assay with MDCK cells infected with B/Malaysia/2506/2004, KL-BHA-4C2 induced robust activity, reaching approximately 30-fold above the anti-H6 irrelevant IgG control. In contrast, KL-BHA-3A7 did not measurably induce ADCC.

Since antibodies binding head-distal HA surfaces can inhibit neuraminidase activity through steric hindrance, we next tested whether these mAbs affect neuraminidase function. Neuraminidase inhibition was measured by enzyme-linked lectin assay (ELLA) using B/Lee/1940 virus on fetuin-coated plates (Fig. 2C). At the highest concentration tested (50 µg/mL), several mAbs showed measurable neuraminidase inhibition. KL-BHA-4C2 and KL-BHA-3D7 were the most active, each inhibiting neuraminidase by ~70%–80%. Notably, KL-BHA-3D7 showed strong neutralization and neuraminidase-inhibition activity against B/Lee/1940 despite its comparatively weak apparent binding in the whole-virion ELISA, further demonstrating that the functional activities of these antibodies do not correlate directly with the ELISA-derived minimum binding concentrations.

### Structural basis for the distinct antiviral activities of influenza B virus HA antibodies

To define the structural basis for the distinct functional profiles observed in the neutralization, viral egress, neuraminidase inhibition, and ADCC assays, we determined the cryo-EM structures of influenza B virus-specific mAbs KL-BHA-3F4, KL-BHA-3D7, and KL-BHA-4C2 in complex with influenza B virus HA (Fig. 3; Table S1). The structures revealed that mAb KL-BHA-3F4 (Fig. 3A) engaged the viral RBS, whereas KL-BHA-3D7 and KL-BHA-4C2 recognized partially overlapping epitopes within a conserved surface spanning the lateral interface between the lower HA head and upper stem, which we refer to here as the medial junction because of its resemblance to an epitope on group 1 influenza A virus HAs previously defined by antibody 2-2-1G06 (Fig. 3E through L; Fig. S5) (35).

**Fig 3:**
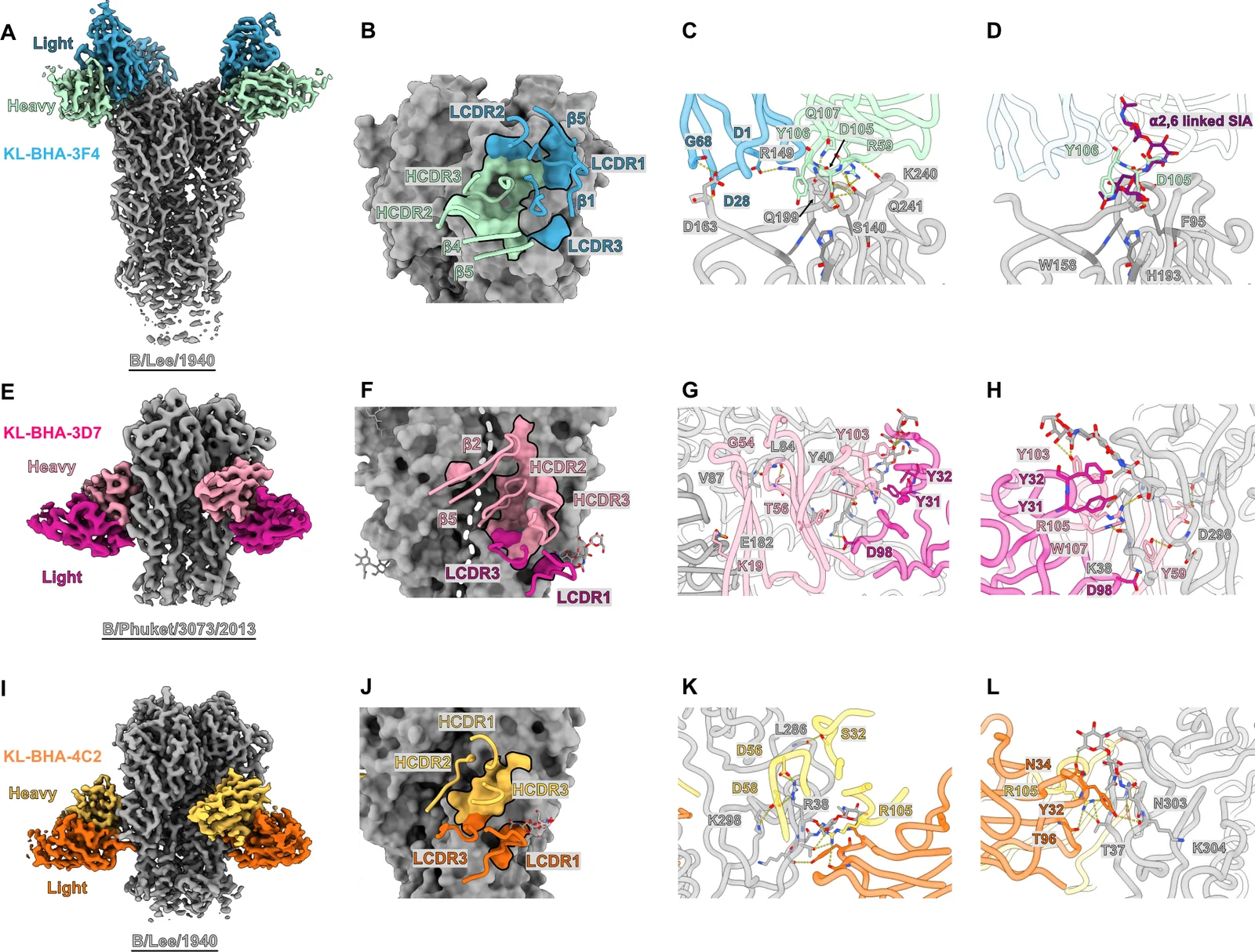
Cryo-EM structures of influenza B virus HA in complex with monoclonal antibodies reveal distinct modes of recognition. (**A**) Cryo-EM map of KL-BHA-3F4 Fab (light chain, blue; heavy chain, light green) in complex with B/Lee/1940 HA at 2.42 Å resolution. (**B**) Footprint of KL-BHA-3F4 on the HA surface with major contacting CDRs shown in loop representation. (**C**) Detailed view of KL-BHA-3F4:HA interactions, with hydrogen bonds shown as dashed yellow lines and interacting residues labeled. (**D**) Heavy chain residues Y106 and D105 mimic interactions of α2,6-linked sialic acid, shown by overlay with PDB ID 4NRK. (**E**) Cryo-EM map of KL-BHA-3D7 Fab (light chain, magenta; heavy chain, pink) in complex with B/Phuket/3073/2013 HA at 3.96 Å resolution. (**F**) Footprint of KL-BHA-3D7 on the HA surface, with protomer boundaries indicated to highlight contacts spanning adjacent protomers. (**G**) Detailed view of KL-BHA-3D7:HA interactions, with hydrogen bonds shown as dashed yellow lines. (**H**) Heavy chain Y103 and VL Y31/Y32 interact with the N303 glycan. (**I**) Cryo-EM map of KL-BHA-4C2 Fab (light chain, orange; heavy chain, yellow) in complex with B/Lee/1940 HA at 2.61 Å resolution. (**J**) Footprint of KL-BHA-4C2 on the HA surface with major contacting CDRs shown. (**K**) Detailed view of KL-BHA-4C2:HA interactions, with hydrogen bonds shown as dashed yellow lines. (**L**) Light chain residues Y32 and N34 interact with the N303 glycan. HA residue numbering throughout the figure follows the mature B/Lee/1940 HA sequence and excludes the signal peptide.

The high-resolution cryo-EM volume of the KL-BHA-3F4:B/Lee/1940 HA complex shows a 1:1 Fab:HA stoichiometry with the Fab variable heavy and light domains “plunging into” the viral RBS at a ~45° angle relative to the HA central 3-fold symmetry axis (Fig. 3; Fig. S2).

The KL-BHA-3F4 antigen-combining site, burying ~1,058 Å^2^ surface area, includes contributions from both heavy and light-chain complementarity-determining regions (CDRs), including HCDR3, HCDR2, and all three LCDR loops (Fig. 3B). While the interaction is dominated by insertion of the HCDR3 loop into the RBS cavity, additional heavy chain contacts are provided by HCDR2 loop interacting with the HA 240-loop, and minor interactions of the heavy framework (FR) 3 strands β4 and β5 engaging the HA 140-loop (Fig. 3B). The HCDR3 residues D105 and Y106 recapitulate key contacts typically formed by the sialic acid receptor. Specifically, the D105 side chain provides the acidic carboxyl group interacting with S140 and Q241 within the RBS, whereas the Y106 aromatic side chain stacks against W158 (Fig. 3C). The two HCDR3 residues closely mimic the sialic acid contacts. Indeed, superposition with α2,6-linked sialic acid demonstrates that KL-BHA-3F4 D105 mimics the carboxylate and the Y106 the acetamido moieties of sialic acid (Fig. 3D). Additional contacts from the LCDR1 and LCDR2 engage the HA 160 loop and the 190 helix, respectively, contributing to a relatively large antibody footprint (Fig. 3B and C). Thus, insertion of the KL-BHA-3F4 HCDR3 into the RBS provides a structural explanation for its hemagglutination-inhibition activity and its ability to interfere directly with receptor engagement.

The other two neutralizing antibodies, KL-BHA-3D7 and KL-BHA-4C2, in contrast, bind the HA more proximally to the viral membrane (Fig. 3E through L).

KL-BHA-3D7 also targets the HA head but does not engage the RBS. Rather, the mAb binds a medial-junction epitope on the lateral surface formed by the vestigial esterase domain and the C-terminus of the HA1 subdomain (Fig. 3; Fig. S3). Although engaging a different set of amino acid residues on HA, KL-BHA-3D7 would compete with mAb 5A7, whose epitope was mapped by mutagenesis (36). Binding is mediated primarily by HCDR2 and HCDR3, with additional contributions from LCDR1 and LCDR3 (Fig. 3F), generating a buried surface area of ~1,357 Å^2^. Heavy-chain residues Y31, Y32, and D98, together with light-chain residues Y103, R105, and W107, form a network of hydrogen bonds and close packing interactions with conserved HA residues within the medial junction (Fig. 3G and H). Notably, many of these contacts involve HA main-chain carbonyls, providing a structural explanation for the broad binding breadth of this antibody. The KL-BHA-3D7 footprint also extends minimally onto an adjacent HA protomer through a salt bridge between heavy-chain residue K19 and HA residue E182 (Fig. 3F). Although this interaction establishes a cross-protomer contact, its functional contribution to antibody binding or inhibition remains unclear. The RBS-distal, quaternary binding mode of KL-BHA-3D7 is therefore consistent with its lack of HAI activity and provides a structural rationale for its predominantly post-entry antiviral activity and inhibition of neuraminidase function through steric interference at the virion surface.

In contrast, KL-BHA-4C2 binds more proximally to the viral membrane (Fig. 3; Fig. S4) and approaches the HA with its main axis perpendicular to that of the HA 3-fold symmetry axis, with an angle of approach that facilitates contacts primarily mediated by all three HCDRs, with additional contributions from LCDR1 and LCDR3 (Fig. 3J). The interaction, burying a relatively small ~648 Å^2^ surface area, is centered on an adjacent conserved surface within the medial junction, where antibody heavy chain residues D56 and D58 form salt bridges with HA R38 and K298, respectively, and R105 forms a hydrogen bond with T37 on HA. The light chain residue T96, through its main chain carbonyl, forms polar contacts through hydrogen bonding with T37 on HA, while the light chain Y32 engages the main chain carbonyl on HA K304 residue. Additional contacts are made by the light chain buttressing the HA N303 glycan through Y32 and N34 LCDR1 (Fig. 3J through L). These interactions are contained within a single HA protomer. Thus, although KL-BHA-3D7 and KL-BHA-4C2 target overlapping regions of the HA, KL-BHA-3D7 engages a partially distinct footprint, revealing an alternative mode of recognition of conserved HA epitopes. KL-BHA-3D7 also recognizes the N303 glycan on HA, with the main interactions including aromatic stacking of the heavy chain Y103 and the light chain Y32. The medial-junction epitope targeted by KL-BHA-4C2 similarly provides a structural context for its neuraminidase-inhibitory activity, while its strong ADCC activity indicates that protection by this antibody is not dependent on potent direct neutralization.

Together, these structures explain the distinct functional profiles of the antibodies. KL-BHA-3F4 directly engages and mimics the receptor within the RBS, whereas the medial-junction binding modes of KL-BHA-3D7 and KL-BHA-4C2 position the antibodies near the virion surface and provide structural context for their neuraminidase-inhibitory activity. KL-BHA-3D7 additionally exhibits post-entry antiviral activity, whereas KL-BHA-4C2 mediates robust Fc-dependent effector activity.

### Broadly reactive antibodies target conserved sites of vulnerability on influenza B virus HA

We next compared the footprints of KL-BHA-3F4, KL-BHA-3D7, and KL-BHA-4C2 with sequence conservation across influenza B virus HA and with previously described broadly reactive antibodies (Fig. 4). We mapped each footprint onto the HA surface colored by conservation across representative HAs derived from ancestral, B/Victoria/2/1987-like, and B/Yamagata/16/1988-like viruses and found that all three antibodies target conserved regions of the HA glycoprotein. KL-BHA-3D7 and KL-BHA-4C2 both recognized conserved surfaces on the HA, with KL-BHA-3D7 and KL-BHA-4C2 binding partially overlapping but distinct epitopes within the medial junction (Fig. 4A, B, and D), consistent with the distinct binding modes observed in the cryo-EM reconstructions and the differences observed in functional assays. We then overlaid these epitopes with those of the previously described influenza B virus antibodies CR8071 and CR9114. KL-BHA-3D7 showed extensive overlap with CR8071, but not with CR9114 (Fig. 4E and G). Although the KL-BHA-3D7 footprint overlapped with that of CR8071, it extended lower and more laterally on the HA surface, defining a distinct medial-junction epitope rather than a canonical vestigial esterase epitope. KL-BHA-3D7 also makes a limited contact with a neighboring HA protomer through a salt bridge between antibody heavy-chain residue K19 and HA residue E182. In contrast, KL-BHA-4C2 only bound a single HA protomer and partially overlapped with CR8071 and, to a lesser extent, with CR9114 (Fig. 4H and I), further supporting the conclusion that these two antibodies achieve breadth through distinct structural solutions. We performed a similar comparison for the head-directed antibody KL-BHA-3F4 and found that its footprint closely matched that of the previously described human RBS antibody H2365 (Fig. 4C and F). Together, these analyses show that the antibodies characterized here occupy highly conserved antigenic surfaces on influenza B virus HA and place KL-BHA-3F4, KL-BHA-3D7, and KL-BHA-4C2 within a broader structural landscape of RBS- and medial-junction-directed broadly reactive antibodies.

**Fig 4:**
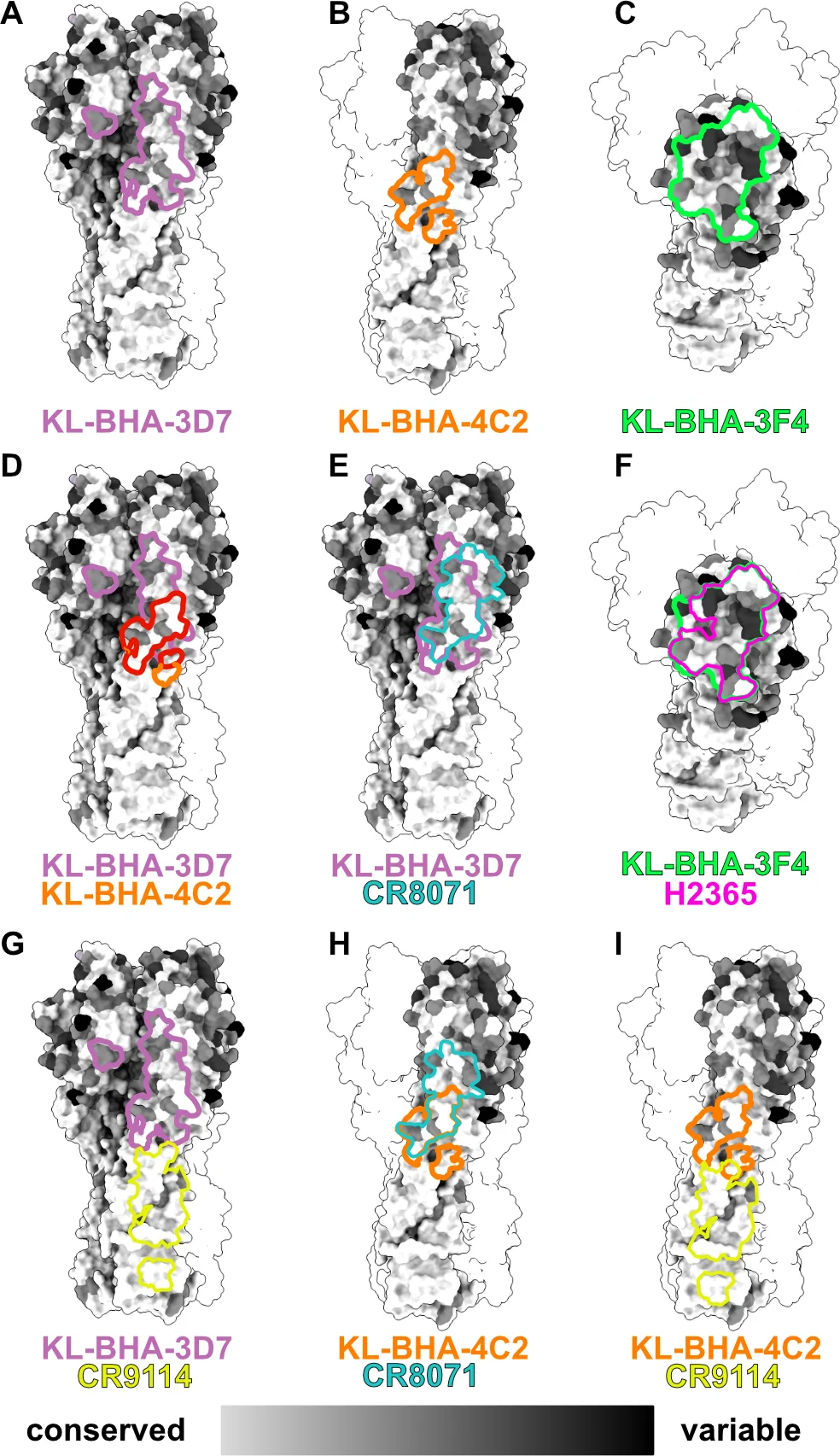
Footprints of KL-BHA-3D7, KL-BHA-4C2, and KL-BHA-3F4 and other broadly binding mAbs mapped onto sequence conservation of influenza B virus HA. Surface representation of influenza B virus HA colored by sequence conservation across representative influenza B viruses (as in Fig. 1A), from conserved (pale gray) to variable (dark gray). Antibody footprints are outlined on the HA surface. (**A**) KL-BHA-3D7 footprint within the HA medial junction. (**B**) KL-BHA-4C2 footprint within the HA medial junction. (**C**) KL-BHA-3F4 footprint on the HA head at the receptor-binding site. (**D**) Overlay of KL-BHA-3D7 and KL-BHA-4C2 footprints showing partially overlapping but distinct medial-junction epitopes. (**E**) Overlay of KL-BHA-3D7 and the previously described influenza B virus antibody CR8071. (**F**) Overlay of KL-BHA-3F4 and the previously described receptor-binding site antibody H2365. (**G**) Overlay of KL-BHA-3D7 and the central-stem antibody CR9114. (**H**) Overlay of KL-BHA-4C2 and CR8071. (**I**) Overlay of KL-BHA-4C2 and CR9114.

### The mAbs confer protection against lethal influenza B virus challenge *in vivo*

Having defined the functional profiles, binding modes, and conserved epitopes of these antibodies, we evaluated whether these distinct antiviral mechanisms conferred protection *in vivo* using a lethal influenza B virus challenge model (Fig. 5). BALB/c mice received antibodies intraperitoneally (IP) at 5 mg/kg and were challenged with 5 × 50% murine lethal dose (mLD50) of B/Malaysia/2506/2004. When administered prophylactically 2 h before infection, all four mAbs conferred complete protection, resulting in 100% survival (Fig. 5A and B). Mice treated with KL-BHA-3F4 or KL-BHA-4C2 exhibited minimal weight loss, whereas those receiving KL-BHA-3A7 lost approximately 15% of body weight before recovery (Fig. 5A). In contrast, all mice treated with the negative control anti-H6 mAb 8H9 succumbed to infection by day 9.

**Fig 5:**
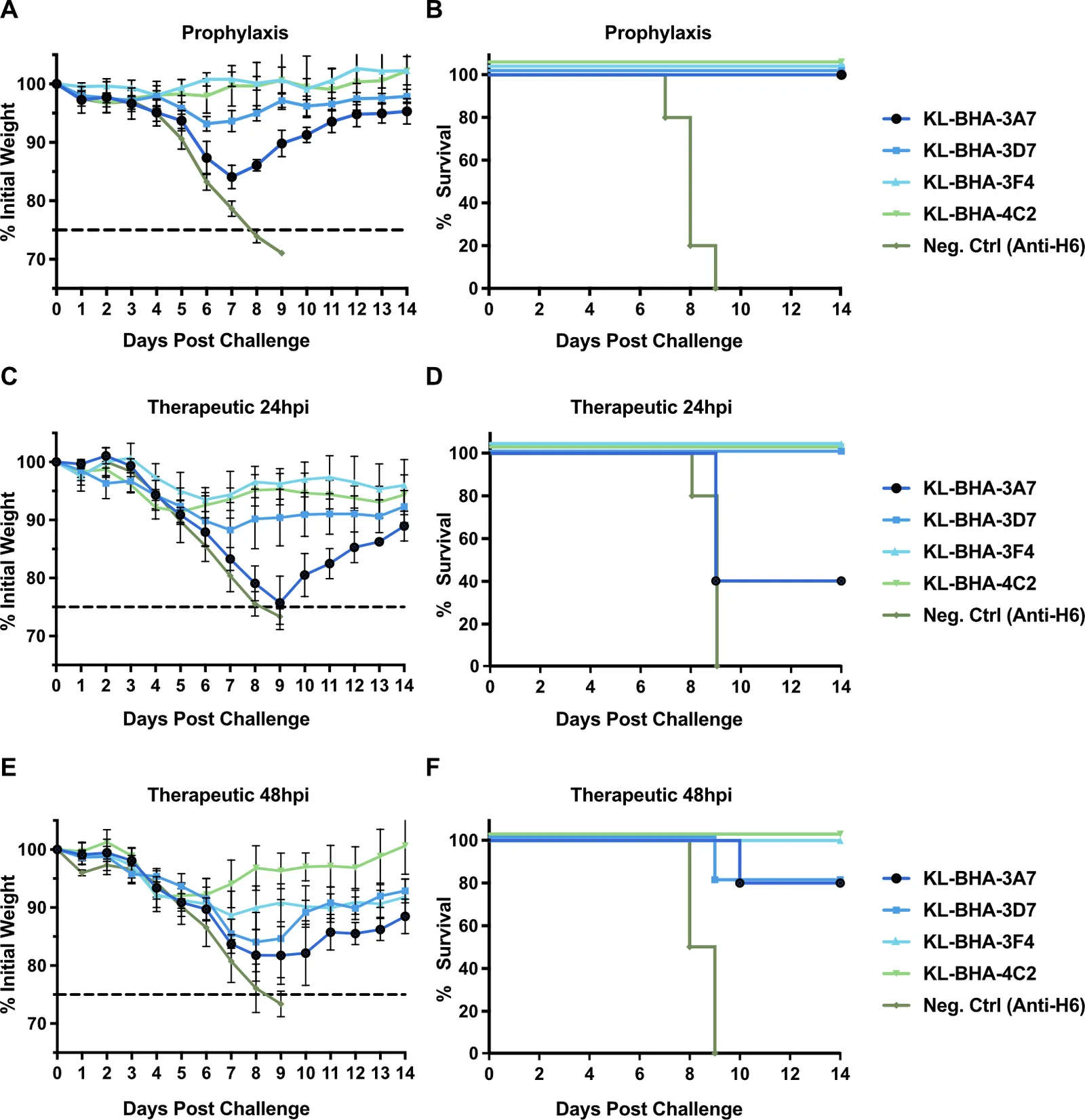
Anti-influenza B virus HA monoclonal antibodies confer protection against lethal viral challenge in mice. Mice (*n* = 5 per group) received mAbs (5 mg/kg, intraperitoneally) and were challenged intranasally with 5 × mLD₅₀ of influenza B/Malaysia/2506/2004. Antibodies were administered (**A** and **B**) prophylactically 2 h before infection or therapeutically at (**C** and **D**) 24 h or (**E** and **F**) 48 h after infection. Body weight was monitored for 14 days, and the mean weight loss ± SD is shown. Kaplan-Meier survival curves are presented for the same period. The dashed line indicates the 25% weight loss threshold for euthanasia.

We next assessed therapeutic efficacy by administering the mAbs at 24 or 48 h after infection (Fig. 5C through F). KL-BHA-3A7 provided little therapeutic benefit, with weight loss profiles resembling those of the negative control group at both time points. In contrast, mice treated with KL-BHA-4C2 began to recover as early as day 4 after infection and were fully protected from virus-induced mortality when the antibody was administered at either 24 or 48 h. Mice receiving KL-BHA-3D7 at 24 or 48 h after infection showed weight loss kinetics similar to those observed with prophylactic administration through day 6, consistent with substantial therapeutic activity.

## DISCUSSION

In this study, we defined complementary structural and functional solutions by which antibodies can achieve broad activity against influenza B virus HA. The four antibodies examined here retained binding across influenza B virus isolates spanning nearly eight decades and both major antigenic lineages. Despite extensive binding breadth, two of the four antibodies, KL-BHA-3A7 and KL-BHA-4C2, showed a modest neutralization profile, while the other two, KL-BHA-3F4 and KL-BHA-3D7, neutralized broadly. Our further in-depth functional analyses also showed that antibodies partitioned into mechanistically distinct classes. KL-BHA-3F4 acted as a receptor-blocking antibody with clear hemagglutination inhibition activity. KL-BHA-3D7 lacked HAI activity and acted predominantly during the post-entry phase, in addition to inhibiting neuraminidase activity. In contrast, KL-BHA-4C2 showed no detectable entry- or egress-inhibitory activity but mediated substantial neuraminidase inhibition and robust Fc-dependent effector activity. KL-BHA-3A7 showed only modest activity in any of these assays. Together, these data support the view that broad protection against influenza B virus need not be restricted to a single neutralization mechanism but can instead arise through multiple modes of engagement of the HA glycoprotein.

We determined the cryo-EM structures for three of the four antibodies and contributed to a structural understanding of how these distinct antibody neutralization mechanisms map onto antigenic space on influenza B virus HA. KL-BHA-3F4 engages the RBS through insertion of HCDR3 residues that mimic key contacts made by sialic acid, explaining its potent hemagglutination inhibition activity and potent neutralization. This binding mode is reminiscent of previously described human RBS-directed antibodies, which use a dipeptide motif at the tip of HCDR3 to reproduce critical chemical features of receptor engagement (28, 37). In a similar manner, murine mAb KL-BHA-3F4 uses D105 and Y106 to mimic the sialic acid carboxylate and acetamido interactions within the RBS, indicating convergence on a common structural solution despite differences in sequence composition and species origin. This convergence suggests that the influenza B virus HA RBS is shaped by conserved structural constraints that position receptor mimicry as an effective mode of neutralization and further supports the RBS as a vulnerable site that can be targeted across diverse antibody repertoires. KL-BHA-3F4 also showed activity when added after initial infection. This result does not necessarily indicate direct inhibition of egress because potent receptor blockade could prevent newly released virions from attaching to neighboring cells and initiating subsequent rounds of infection. The moderate neuraminidase-inhibitory activity of KL-BHA-3F4 may provide an additional contribution through steric interference with NA function.

KL-BHA-3D7 and KL-BHA-4C2, by contrast, bind partially overlapping but distinct conserved epitopes within the medial junction and do so with distinct footprints and geometries. KL-BHA-4C2 recognizes a compact epitope within a single protomer and engages the N303 glycan. KL-BHA-3D7 also engages the N303 glycan and makes a limited cross-protomer contact through a single salt bridge between antibody heavy-chain residue K19 and HA residue E182. The functional significance of this interaction remains uncertain. These differences likely underlie the functional divergence between the two antibodies, including the stronger neuraminidase inhibition and therapeutic activity observed for KL-BHA-3D7 and the potent ADCC activity observed for KL-BHA-4C2. The findings therefore extend the concept that conserved influenza virus HA epitopes can be recognized by more than one productive structural solution, even within the same general HA epitopic region.

All four antibodies bound broadly; however, their antiviral activities separated into distinct profiles depending on whether the readout emphasized receptor blockade, plaque reduction, viral egress, neuraminidase inhibition, or Fc-mediated effector function. This was especially evident for KL-BHA-4C2, which showed modest neutralization in plaque reduction assays against some strains but substantial neuraminidase inhibition, strong ADCC activity, and complete protection *in vivo*, including therapeutic efficacy. Conversely, KL-BHA-3A7 exhibited broad binding but comparatively limited activity in several functional assays and little therapeutic benefit. These differences indicate that conserved epitope recognition can support protection through qualitatively different mechanisms, and that evaluation of influenza B virus antibodies may require a broader set of functional assays that extend beyond neutralization alone.

The apparent binding strength measured by whole-virion ELISA did not consistently predict HAI, neutralization, or neuraminidase-inhibition activity. This likely reflects the distinct properties measured by each assay. The minimum binding concentration obtained by whole-virion ELISA is a semiquantitative measure influenced by avidity, antigen density, virion adsorption, and epitope accessibility rather than a direct measurement of intrinsic antibody affinity. HAI specifically measures interference with HA-receptor engagement, whereas plaque-reduction assays integrate effects on multiple stages of the viral life cycle. Similarly, neuraminidase inhibition by an HA-directed antibody depends on the position and geometry of the bound antibody relative to NA. These differences may explain why KL-BHA-3D7 showed strong neutralization and neuraminidase inhibition against B/Lee/1940 despite comparatively weak apparent binding by ELISA. Strain-specific differences in HA and NA abundance or organization, glycosylation, receptor avidity, and HA-NA functional balance may also contribute, although these possibilities were not directly tested in this study.

The results also have implications for influenza B virus immunogen design. Much of the effort to develop broader influenza vaccines has focused on redirecting immunity toward conserved HA surfaces, particularly in the stem. The current data suggest that this goal may be worthwhile for influenza B virus, but that “stem immunity” should not be treated as a single category. The medial-junction antibody KL-BHA-4C2 is distinct from canonical central-stem antibodies and combines neuraminidase inhibition with Fc-dependent effector activity. Thus, for influenza B virus, the desirable response may not simply be enrichment of any stem-specific antibody, but preferential induction of structural classes that combine breadth with mechanisms most likely to be protective *in vivo*. At the same time, the identification of an RBS-directed antibody with broad activity across ancestral, B/Victoria/2/1987-like, and B/Yamagata/16/1988-like viruses indicates that the conserved RBS also remains an accessible target and may be a valuable component of a broader vaccine strategy.

Finally, for vaccine translation, the challenge will be to design immunogens that reproducibly elicit favorable classes of antibodies rather than merely broad binding. Some such strategies, including HA resurfacing (25), hyperglycosylation (24), and chimeric or mosaic HA (34, 38–41) generation, have been applied for influenza A virus HA, but research on influenza B has been lagging. These efforts will likely require iterative integration of structural epitope definition, immune focusing strategies, and functional benchmarking in relevant models.

Several limitations should be considered when interpreting our findings. First, the antibodies were generated in mice after sequential exposure to selected influenza B virus antigens, and the resulting repertoires may not fully reflect the ontogeny, clonal selection, or structural solutions favored in human infection or vaccination. Second, the *in vivo* protection studies relied on a single mouse model and primarily one challenge virus, which limits inference about protection across hosts, viral backgrounds, and disease settings.

The *in vivo* studies assessed protection through survival and weight loss but did not measure pulmonary viral burden. Therefore, whether antibodies with distinct functional profiles differ in their effects on viral replication or clearance remains unresolved.

Finally, the observed therapeutic benefit will require cautious interpretation because murine Fc receptor biology, antibody pharmacokinetics, and the timing of intervention differ from those in humans. These considerations place appropriate boundaries on how directly the present results can be extrapolated to clinical performance in humans.

From a broader perspective, our findings support a view of influenza B virus HA as a mosaic of conserved functional surfaces that can be productively targeted by antibodies with different structural solutions and different modes of antiviral activity. For intervention strategies, this raises the possibility that the most effective protection may come not from maximizing one antibody type but from combining complementary mechanisms including receptor blockade, locking HA in pre-fusion conformation, interference with viral egress, and Fc-mediated clearance within a single therapeutic cocktail or vaccine-elicited response.

## MATERIALS AND METHODS

### Cells, viruses, and recombinant proteins

MDCK cells (ATCC #CCL34) were cultured in complete Dulbecco’s modified Eagle medium (DMEM, Life Technologies) supplemented with Pen-Strep (100 U/mL penicillin and 100 µg/mL streptomycin; Gibco), 1 M 4-(2-hydroxyethyl)-1-piperazineethanesulfonic acid (HEPES; Life Technologies), and 10% fetal bovine serum (FBS; Hyclone). Sf9 (ATCC #CRL1711) and High Five cells (BTI- TN-5B1-4 subclone; Vienna Institute of Biotechnology) were propagated in *Trichoplusia ni* medium-formulation Hink insect medium (TNM-FH; Gemini Bioproducts) supplemented with 10% FBS and Pen-Strep, and serum-free Sf-900 II medium (Gibco), respectively. The hybridoma clones were cultured initially in ClonaCell-HY Medium E (Stemcell Tech) and progressively transitioned to Hybridoma serum-free medium (Hybridoma-SFM; Life Technologies). Expi293F (Gibco #A14527) cells were cultured in Expi293 media as per the manufacturer’s instructions. B/Lee/1940, B/Victoria/2/1987 (V), B/Malaysia/2506/2004 (V), B/New York/PV01181/2018 (V), B/Yamagata/16/1988 (Y), B/Florida/4/2006 (Y), and B/New York/PV00094/2017 (Y) viruses were propagated in 10-day-old embryonated chicken eggs (Charles River Laboratories) incubated at 33°C for 72 h. Allantoic fluid was harvested and spun at 2,000 × *g* for 10 min at 4°C to remove cellular debris. To generate purified virions, ultracentrifugation at 25,000 rpm at 4°C for 2 h was carried out with a 30% wt/vol sucrose cushion in NTE buffer (100 mM NaCl, 10 mM Tris-HCl, and 1 mM ethylenediaminetetraacetic acid [EDTA]; pH 7.4). The pellet was resuspended in phosphate-buffered saline (PBS; Gibco). Any recombinant HAs used in the screening of the mAbs following hybridoma fusions were generated as previously described (42, 43). Antibodies were produced using a hybridoma fusion-based approach as described in detail previously (33). Briefly, a 6- to 8-week-old female BALB/c mouse was sublethally infected with B/Lee/1940 and B/Malaysia/2506/2004 (V) 4 weeks apart, followed by IP injection of the purified A/Puerto Rico/8/1934 (PR8) virus expressing B/Yamagata/16/1988 (Y) HA 4 weeks later (44). Recombinant HAs were expressed and purified from High Five cell culture supernatant as described previously (42). HA residues are numbered according to the mature B/Lee/1940 HA sequence, beginning with the N-terminal DRI residues and excluding the signal peptide. Residues in other influenza B virus HA strains were assigned by sequence alignment and are reported using the corresponding B/Lee/1940 numbering.

Splenocytes from the mouse’s spleen were fused with SP2/0 myeloma cells, following which clones propagated in semisolid ClonaCell-HY medium D (Stemcell Technologies) were expanded in 96-well plates and grown in medium E. The supernatants were screened against a variety of different influenza B purified viruses and recombinant HAs. The mAbs were isotyped using a Pierce rapid antibody isotyping kit (Life Technologies). For KL-BHA-4C2, the hybridoma clone was expanded in Hybridoma-SFM media. Due to issues with cryostock recovery, the variable regions of KL-BHA-3A7, KL-BHA-3D7, and KL-BHA-3F4 were sequenced and cloned into pFUSEss-CHIg and pFUSEss-CLIg (Invivogen) into the corresponding isotype backbone. The heavy and light chain plasmids were transiently transfected and expressed in Expi293F cells using the Expi293 expression system (Gibco) as per the manufacturer’s instructions. All antibodies were purified on an ÄKTA pure chromatography system (GE Healthcare) with a HiTrap HP Protein G column (Cytiva Life Sciences).

### ELISAs

Immulon 4HBX microtiter flat-bottom plates (Thermo Scientific) were coated with 5 µg/mL of purified virus or 2 µg/mL of recombinant HA (cH8/B) in 1× KPL coating solution (SeraCare) overnight at 4°C. The plates were washed 3× with PBS containing 0.1% Tween 20 (PBST) and blocked with 200 µL of blocking buffer comprising PBST, 3% goat serum (Gibco), and 0.5% dry non-fat milk (American Bio) per well for 1 h at room temperature (RT); mAbs were diluted 3-fold starting at 30 µg/mL in blocking buffer and added to the plates after discarding the blocking buffer. The plates were incubated for 2 h at RT and washed three times with PBST using an Aquamax 4000 microplate washer device (Molecular Devices). Each well received 50 µL anti-mouse horseradish peroxidase (HRP)-labeled antibody (Rockland) diluted to 1:3,000 in blocking buffer, and the plate was incubated at RT for 1 h. The plates were washed four times with PBST, shaking with the plate washer, and 100 µL of SigmaFast o-phenylenediamine dihydrochloride (OPD; Sigma-Aldrich) was added to each well. The plates were developed for 10 min, and 50 µL of 3 M hydrochloric acid was added to each well to stop the reaction. Optical density (OD) at 490 nm was determined using a Synergy H1 microplate reader (BioTek). Minimum binding concentration (MBC) was defined as the final concentration of the mAb in µg/mL at which the OD was above the cut-off value calculated as the mean plus three times the standard deviation of the blank wells’ ODs. Anti-influenza B virus NA KL-BNA-4F11 and anti-H6 8H9 were used as positive and negative controls, respectively (45). The MBCs were visualized as a heatmap generated on RStudio and annotated on Adobe Illustrator.

### Phylogenetic and comparative sequence analysis

Sequences for the tested influenza B viruses were obtained from Global Initiative on Sharing All Influenza Data (GISAID), and multiple sequence alignment was performed on Clustal Omega. For the rendered model highlighting the HA amino acid conservation across the seven tested viruses, UCSF ChimeraX was used (46). The multiple sequence alignment was uploaded to UCSF ChimeraX, and amino acid conservation at each site was represented by superimposition on the publicly available B/Lee/1940 HA structure (PDB: 4NRJ).

### Plaque reduction neutralization assay (PRNA)

The mAbs at a starting concentration of 100 µg/mL were diluted 1:5 six times in 1× minimal essential medium (1× MEM). Approximately 1,000 plaque-forming units (PFU) of each virus were incubated with the mAb dilutions for 1 h at RT, shaking, and 200 µL of this mixture was transferred onto a monolayer of MDCK cells in a 12-well plate after they were washed once with PBS. The plates were incubated at 33°C for 50 min, shaken every 10 min. After the incubation, agar overlays containing 1 μg/mL tosyl phenylalanyl chloromethyl ketone (TPCK)-treated trypsin and corresponding mAb dilutions were added, and the plates were incubated at 33°C for 72 h. The cells were fixed with 3.7% paraformaldehyde for 1 h at RT. The wells were blocked with 3% milk in PBS for 1 h at RT after removing the overlay. The plaques were stained with an anti-influenza B virus HA and NA mAb cocktail for 1 h at RT, washed three times with PBS, and stained with a 1:3,000 dilution of anti-mouse IgG HRP secondary antibody (Rockland). The plates were washed three times again with PBS, and plaques were developed with TrueBlue reagent (KPL). The number of plaques was counted, and percent reduction in plaque number was calculated in comparison to an irrelevant IgG anti-H6 8H9 control. The 50% inhibitory concentration (IC_50_), defined as the concentration at which 50% plaque number reduction is observed, was calculated using a non-linear regression model on GraphPad Prism 7. The heatmap visualizing the IC_50_ values was generated on RStudio and annotated on Adobe Illustrator.

### Hemagglutination inhibition assay

Hemagglutination assay was performed to measure the hemagglutination titer (HAU) for the viruses; mAbs were diluted 2-fold in a V-bottom plate starting at 100 µg/mL in 1× PBS. A 1:1 ratio of 8 HAU/50 µL was added to the mAb dilutions, and the plates were incubated shaking at RT for 30 min. Following this, 50 µL of 0.5% chicken red blood cells (RBCs) diluted in PBS was added to each well, and the plates were incubated at 4°C for an hour. ”No virus” and “no antibody” controls were used on each plate. The well prior to the first well exhibiting hemagglutination was determined to be the minimum HI concentration (µg/mL), and these values were visualized in a heatmap made on RStudio, annotated on Adobe Illustrator.

### Microneutralization assay

Interference in viral entry or egress was assessed using microneutralization assays. MDCK cells were seeded in 96-well tissue culture plates (Corning) at 20,000 cells per well. The subsequent day, mAbs were diluted 2-fold, and B/Lee/1940 virus was diluted to 100 times the 50% tissue culture infectious doses (TCID50)/mL in 1× MEM with 1 µg/mL TPCK-treated trypsin (infection medium). Virus mixed with equal volumes of either mAb dilution or infection medium was incubated together in 96-well plates for 1 h at RT with shaking. A second set of mAb dilutions was prepared in the infection medium. Prior to completion of the 1 h, the cell plates were washed with PBS, and 100 µL from virus + mAb plates was transferred to the corresponding cell plates for “entry only” and “entry and egress” conditions, while the virus incubated with the infection medium was added to the cell plates for the “egress only” condition. The plates were incubated at 33°C for 1 h, after which the inoculum was aspirated, and the cells were washed with PBS. One hundred microliters of corresponding mAb dilutions were added to the “entry and egress” and “egress only” cell plates, while the infection medium was added to the “entry only” cell plates. The plates were then incubated at 33°C for 72 h. To determine the extent of neutralization and virus replication, a hemagglutination assay was carried out in V-bottom 96-well plates with 50 µL of the supernatant and 50 µL of 0.5% chicken RBCs. The well prior to the first well at which hemagglutination was seen, indicating that virus replication was determined to be the minimum neutralization concentration, and these values were visualized in GraphPad Prism 7.

### Neuraminidase inhibition assay

Neuraminidase inhibition assays were performed as described earlier (45). Briefly, 96-well flat-bottom Immulon 4 HBX plates (Thermo Scientific) were coated with 25 µg/mL fetuin overnight at 4°C; mAbs were diluted in Dulbecco’s phosphate-buffered saline (DPBS; Gibco) containing 0.05% Tween 20 and 1% bovine serum albumin (BSA) 2-fold from a starting concentration of 50 µg/mL. B/Lee/1940 virus was diluted to 2× the 50% effective concentration (EC_50_) as determined by an ELLA in DPBS containing Tween 20 and BSA and was added to the mAb dilutions. The plates were shaken and incubated for 90 min at RT. The fetuin-coated plates were washed three times with PBST, and 100 µL of the virus-mAb mixture was transferred into these plates. The plates were sealed and incubated at 33°C for 16–18 h. Following the incubation, the plates were washed six times with PBST, and 100 µL of peanut agglutinin conjugated to HRP (PNA-HRP) at a concentration of 2 µg/mL diluted in DPBS containing 1% BSA was added. After incubating for 2 h at RT in the dark, the plates were washed six times with PBST and developed with 100 µL of SigmaFast OPD. The reaction was stopped after 10 min with 50 µL of 3 M hydrochloric acid, and the absorbance at 490 nm was measured using a Synergy H1 microplate reader (BioTek). Anti-influenza B virus neuraminidase antibody KL-BNA-1F2 was used as a positive control, and KL-AV-1B3, an anti-arenavirus antibody, was used as a negative control (47). “No virus” control and a “virus-only” control were used to normalize 100% and 0% neuraminidase inhibition activity. The absorbance values obtained for the mAbs were normalized accordingly to determine the percent inhibition of neuraminidase activity, and these data were visualized using a non-linear regression model in GraphPad Prism 7.

### ADCC reporter assay

Induction of Fc-mediated effector activity by mAbs was assessed using an *in vitro* ADCC reporter bioassay kit (Promega). MDCK cells were seeded in a white 96-well plate (Corning) at 30,000 cells per well. They were infected with B/Malaysia/2506/2004 virus at a multiplicity of infection (MOI) of 5 in a single-cycle infection. After an overnight infection at 33°C, the media were aspirated, and antibody dilutions were added to the plate at 25 µL/well, diluted in Roswell Park Memorial Institute 1640 (RPMI 1640; Gibco) medium. Each well also received 25 µL of assay buffer (RPMI 1640 with low IgG serum; Promega) and 25 µL of ADCC effector cells at 75,000 cells per well. The plates were incubated at 37°C for 6 h, following which 75 µL of the luciferase assay substrate was added to each well. A Synergy H1 microplate reader (BioTek) was used to determine the luminescence values in each well, and the fold induction over the anti-H6 8H9 negative control was plotted on GraphPad Prism 7.

### *In vivo* mouse challenge experiments

All mouse experiments were conducted in accordance with the Icahn School of Medicine at Mount Sinai Institutional Animal Care and Use Committee (IACUC) guidelines. Female 6- to 8-week-old BALB/c mice (Jackson Laboratories; *n* = 5/group) were injected with 5 mg/kg of sterile-filtered mAb IP. The mAbs were administered 2 h prior to viral challenge for the prophylactic setting and at either 24 or 48 h post-infection for the therapeutic setting. Mice were challenged with 5× mLD_50_ of B/Malaysia/2506/2004 challenge virus. Weight loss was monitored over 14 days, and mice were euthanized if they lost 25% of their initial body weight in accordance with the humane endpoints established by the IACUC protocols. Anti-H6 8H9 was used as a negative control. Weight loss curves and Kaplan-Meier survival plots were generated using GraphPad Prism 7.

### Cryo-EM sample preparation

Recombinant B/Lee/1940 and B/Phuket/3073/2013 HAs were expressed in ExpiSf9 cells (Thermo Fisher) using the baculovirus expression system. HA genes were cloned into the pFastBac vector, and recombinant baculovirus was generated and used to infect ExpiSf9 cells for protein production. Supernatants were harvested 3 days post-infection, clarified by centrifugation at 4,000 × *g* for 20 min, and filtered through a 0.45-μm membrane. Recombinant proteins were purified from the filtered supernatant using TALON metal affinity resin (Takara Bio) and eluted as previously described. Eluted proteins were concentrated using 10 kDa Vivaspin 20 centrifugal concentrators (Sartorius) and further purified by size-exclusion chromatography (ÄKTA) for size separation and buffer exchange. KL-BHA-3F4, KL-BHA-3D7, and KL-BHA-4C2 Fabs were generated by papain digestion (Thermo Fisher) of IgG mAbs. For complex formation, B/Lee/1940 HA was incubated with KL-BHA-3F4 or KL-BHA-4C2 Fabs at protein concentrations of 2 mg/mL and 2.5 mg/mL, respectively, and B/Phuket/3073/2013 HA was incubated with KL-BHA-3D7 Fab at 2.5 mg/mL, using a 1.2-fold molar excess of Fab. All mixtures were incubated for 20 min at RT prior to grid preparation. KL-BHA-3F4 complex was prepared on Ultrathin Carbon on Quantifoil, Orthogonal Array 1.2/1.3 grid (Ted Pella, Inc), while KL-BHA-3D7:HA and KL-BHA-4C2:HA complexes were prepared on UltrAuFoil gold R1.2/1.3 grids (Ted Pella, Inc). Grids were subjected to glow-discharging in a PELCO easiGlow device (air pressure: 0.39 mBar, current: 28 mA, and duration: 25 s) before sample preparation. Immediately before grid preparation, n-octyl-β-D-glucoside (Anatrace) was added to the preformed complex at 0.1% wt/vol final concentration. Next, 3-μL aliquots of KL-BHA-3D7 or KL-BHA-4C2 complexes were applied to the grids and subsequently blotted for 5 s using blot force 0 at 22°C and 85% humidity and then plunge-frozen in liquid ethane using a Vitrobot Mark IV system (Thermo Fisher Scientific). As for the KL-BHA-3F4:HA complex, 3 µL of the sample was applied to the grids and blotted for 3 s at 10°C and 85% humidity and then plunge-frozen in liquid ethane using a Leica EM-GP plunge freezer.

### Cryo-EM data collection

Movies for all complexes were aligned and dose-weighted using patch motion correction in cryoSPARC (versions 4.2.1-4.5.3) (48), and contrast transfer function (CTF) parameters were estimated using Patch CTF. Particles were picked using either template-based or blob picking, followed by extraction with appropriate box sizes (480 pixels for KL-BHA-3F4 and KL-BHA-3D7, and 512 pixels for KL-BHA-4C2) with 4× binning and subjected to multiple rounds of 2D classification to remove poorly aligned particles and junk. For KL-BHA-3F4, an initial model with five classes was generated from 1,809,998 particles, and the best two classes (156,610 particles) were selected for refinement; subsequent homogeneous refinement without imposed symmetry, followed by re-extraction without binning and local and global CTF refinement, yielded a final map at 2.42 Å resolution. For KL-BHA-3D7, an initial model with three classes was generated from 300,904 particles, and the best class (144,016 particles) was selected; non-uniform refinement without imposed symmetry, followed by re-extraction and iterative refinement with CTF optimization, produced a final map at 3.98 Å resolution. For KL-BHA-4C2, an initial model with five classes was generated from 1,359,004 particles, and the best class (498,384 particles) was selected. Non-uniform refinement with imposed C3 symmetry, followed by re-extraction and homogeneous refinement with CTF optimization, resulted in a final map at 2.61 Å resolution. For all data sets, the final maps were sharpened using DeepEMhancer (49), with half-maps from the final refinement, and reported resolutions are based on the gold-standard Fourier shell correlation (FSC) 0.143 criterion.

### Model building and refinement

The DeepEMhancer-sharpened focused maps were used for model building. Initial models for the KL-BHA-3F4 and KL-BHA-4C2 complexes were generated with ModelAngelo (50) and then manually refined in Coot (51). For KL-BHA-3D7, an initial model was generated with AlphaFold3 (52), rigid-body fitted into the cryo-EM map in UCSF ChimeraX (46), and further rebuilt manually in Coot. N-linked glycans were added manually in Coot using the glyco extension. All models were refined by real-space refinement in Phenix and validated with MolProbity. Structural biology software and computing environments used in this project were compiled and configured by the SBGrid Consortium (53).

## Data Availability

The EM maps have been deposited in the Electron Microscopy Data Bank (EMDB) under accession codes EMD-76453, EMD-76454, and EMD-76455, for KL-BHA-3F4, 4C2, and 3D7, respectively, and the accompanying atomic coordinates in the Protein Data Bank (PDB) are under accession codes 12ID (3F4), 12IE (4C2), and 12IF (3D7). The raw movies were deposited for 3F4 (EMPIAR-13469) and 4C2 (EMPIAR-13470) and aligned micrographs for 3D7 (EMPIAR-13471). The heavy- and light-chain variable-region sequences of KL-BHA-3A7, KL-BHA-3D7, KL-BHA-3F4, and KL-BHA-4C2 have been deposited in GenBank under accession numbers PZ737698–PZ737705.

## SUPPLEMENTAL MATERIAL

Supplemental material jvi.00802-26-s0001.docx Fig. S1 to S5; Tables S1 to S3.

ASM does not own the copyrights to Supplemental Material that may be linked to, or accessed through, an article. The authors have granted ASM a non-exclusive, world-wide license to publish the Supplemental Material files. Please contact the corresponding author directly for reuse.
