# Supplementary material for: Broadly reactive antibodies against influenza B virus hemagglutinin neutralize and protect through distinct structural mechanisms
Source: J Virol. 2026 Sep 4;100(10):e00802-26. doi: 10.1128/jvi.00802-26 (PMC13712735; doi:10.1128/jvi.00802-26)
Supplement: Supplementary file 1 [file jvi.00802-26-s0001.docx]

**Supplemental Information**

Broadly reactive antibodies against influenza B hemagglutinin neutralize and protect through distinct structural mechanisms

**Authors:** Disha Bhavsar^1,2 †^, Alesandro Civljak^1, †^, Bruno Bonnettaz^1^, Guha Asthagiri Arunkumar^1,^ ^‡^, Florian Krammer^1,2,3,4,5, *^, Goran Bajic^1, *^

**Affiliations:**

¹ Department of Microbiology, Icahn School of Medicine at Mount Sinai, New York, NY, USA

^2^ Center for Vaccine Research and Pandemic Preparedness, Icahn School of Medicine at Mount Sinai, New York, NY, USA
^3^ Department of Pathology, Molecular and Cell-Based Medicine, Icahn School of Medicine at Mount Sinai, New York, NY, USA
^4^ Ignaz Semmelweis Institute, Interuniversity Institute for Infection Research, Medical University of Vienna, Vienna, Austria
^5^ Ludwig Boltzmann Institute for Science Outreach and Pandemic Preparedness, Medical University of Vienna, Vienna, Austria

*Corresponding authors. Email: [florian.krammer@mssm.edu](mailto:florian.krammer@mssm.edu), (F.K.), [goran.bajic@mssm.edu](mailto:goran.bajic@mssm.edu) (G.B.)

† These authors contributed equally to this work

‡ Current address: Moderna Tx – 325 Binney Street, Cambridge, MA – 02142

**Fig. S1.** (**A**) **Binding of the mAbs to a recombinant chimeric cH8/B.** KL-BHA-3A7 and KL-BHA-4C2 showed binding to a cH8/B HA construct. Binding was assessed using ELISAs against recombinant cH8/Bala (two alanines at the head-stalk interface akin to influenza B virus HAs). Anti-H8 head domain mAb, KL-H8-1A7, and anti-influenza B virus NA mAb, KL-BNA-4F11, were used as a positive and negative control, respectively. (**B**) **Neutralization of influenza B viruses by four anti-HA monoclonal antibodies.** Antiviral activity was measured by plaque reduction neutralization assay. Curves show the percentage reduction in plaque number for each virus at five-fold serial dilutions of the indicated mAbs starting at 100 µg/ml, normalized to plaque numbers obtained with the irrelevant anti-H6 IgG control 8H9 at corresponding concentrations.

**Fig. S2. Cryo-EM pipeline for determining the structure of B/Lee1940 HA in complex with KL-BHA-3F4 Fab.**

**Fig. S3. Cryo-EM pipeline for determining the structure of B/Lee1940 HA in complex with KL-BHA-3D7 Fab.**

**Fig. S4. Cryo-EM pipeline for determining the structure of B/Lee1940 HA in complex with KL-BHA-4C2 Fab.**

**Fig. S5. Comparison KL-BHA-3D7 and KL-BHA-4C2 with known medial-junction antibodies.** Footprints are outlined on the surface of B/Brisbane/60/2008 or A/Singapore/1/1957. Representation of the main axis of Fab (A) 2-2-1G06 (blue) and (B) CR8071 (green), KL-BHA-4C2 (yellow) and KL-BHA-3D7 (pink) to represent general approach of the epitope. Comparison of footprint (C) and binding (D) of influenza A mAb 2-2-1G06 (PDB 8TP9) bound to H2 and CR8071 (PDB 4FQJ), KL-BHA-4C2 (12IE) and KL-BHA-3D7 (12IF) bound to influenza B hemagglutinin (PDBs 4FQM).

| **Fab:HA complex** | | | **KL-BHA-3F4:**  **B/Lee/1940** | **KL-BHA-4C2:**  **B/Lee/1940** | **KL-BHA-3D7:**  **B/Phuket/3073/2013** |
| --- | --- | --- | --- | --- | --- |
| **Model** | **Deposition ID** | | **EMD-76453; PDB 12ID** | **EMD-76454; PDB 12IE** | **EMD-76455; PDB 12IF** |
|  | **Composition** | Chains | 12 | 15 | 10 |
|  |  | Atoms (Hydrogens: 0) | 14646 | 15246 | 12735 |
|  |  | Residues – Protein | 1875 | 1942 | 1633 |
|  |  | Ligands | BMA: 3, NAG: 14 | NAG: 16 | BMA: 3, NAG: 14 |
|  | **Bonds (RMSD)** | Length (Å) (# > 4σ) | 0.006 (0) | 0.004 (0) | 0.006 (0) |
|  |  | Angles (°) (# > 4σ) | 1.163 (3) | 0.678 (0) | 1.154 (1) |
|  | **Quality** | MolProbity score | 1.12 | 1.08 | 1.11 |
|  |  | Clash score | 1.13 | 1.42 | 1.11 |
|  | **Ramachandran plot (%)** | Outliers | 0 | 0 | 0 |
|  |  | Allowed | 4.22 | 3.29 | 4.22 |
|  |  | Favored | 95.78 | 96.71 | 95.78 |
|  | **Rama-Z (RMSD)** | whole | -1.08 (0.18) | -0.45 (0.17) | -1.79 (0.19) |
|  |  | helix | 0.33 (0.31) | 0.55 (0.30) | -0.45 (0.33) |
|  |  | sheet | -0.07 (0.21) | 0.76 (0.22) | -1.41 (0.25) |
|  |  | loop | -1.41 (0.16) | -1.14 (0.16) | -1.26 (0.18) |
|  | **Other** | Rotamer outliers (%) | 0 | 0 | 0 |
|  |  | Cβ outliers (%) | NA | NA | NA |
|  |  | Peptide – Cis proline/general (%) | 6.1/0.0 | 3.3/0.0 | 0.0/0.0 |
|  |  | Peptide – Twisted proline/general (%) | 0.0/0.0 | 0.0/0.0 | 0.0/0.0 |
|  |  | CaBLAM outliers (%) | 2.03 | 1.42 | 2.95 |
|  | **ADP (B-factors)** | Iso/Aniso (#) | 14646/0 | 15246/0 | 12735/0 |
|  |  | Protein min/max/mean | 11.36/98.81/39.53 | 15.28/121.06/43.11 | 61.59/109.92/82.25 |
|  |  | Ligand min/max/mean | 17.44/85.78/53.89 | 30.61/104.23/69.33 | 0.00/134.27/87.94 |
|  | **Occupancy** | Mean | 1 | 1 | 1 |
|  |  | occ = 1 (%) | 100 | 100 | 100 |
|  |  | 0 < occ < 1 (%) | 0 | 0 | 0 |
|  |  | occ > 1 (%) | 0 | 0 | 0 |
| **Data** | **Box** | Lengths (Å) | 135.04, 138.42, 118.16 | 140.93, 130.15, 162.48 | 97.29, 115.06, 138.74 |
|  |  | Angles (°) | 90.00, 90.00, 90.00 | 90.00, 90.00, 90.00 | 90.00, 90.00, 90.00 |
|  | **Resolution** | Supplied Resolution (Å) | 2.3 | 2.4 | 4 |
|  |  | d99 full (Masked/Unmasked) | 2.0/2.1 | 2.1/2.1 | 4.4/4.4 |
|  |  | d model (Masked/Unmasked) | 1.6/1.6 | 1.8/1.8 | 4.2/4.2 |
|  |  | d FSC model 0/0.143/0.5 (Masked) | 1.4/1.5/2.4 | 1.4/1.5/2.3 | 3.8/3.9/4.1 |
|  |  | d FSC model 0/0.143/0.5 (Unmasked) | 1.4/1.5/2.4 | 1.4/1.6/2.7 | 3.9/4.0/4.2 |
|  |  | Map min/max/mean | 0 | 0 | -8.3247688 |
| **Model vs. Data** | | CC (mask) | 0.82 | 0.86 | 0.82 |
|  |  | CC (box) | 0.82 | 0.8 | 0.8 |
|  |  | CC (peaks) | 0.79 | 0.74 | 0.62 |
|  |  | CC (volume) | 0.83 | 0.86 | 0.81 |
|  |  | Mean CC for ligands | 0.68 | 0.66 | 0.7 |

**Table S1.** **Cryo-EM data processing and model validation statistics.**

| **Antibody name** | **CDR3 sequence** | **CDR3 AA length** |
| --- | --- | --- |
| 3A7 heavy chain | CARGAGTIAYW | 11 |
| 3A7 kappa light chain | CLQYDEFPLTF | 11 |
| 3F4 heavy chain | CARHGSIYYDYQGYFDYW | 18 |
| 3F4 kappa light chain | CQQHYSSPWTF | 11 |
| 3D7 heavy chain | CARSGSSYVRDWYFDVW | 17 |
| 3D7 kappa light chain | CQQSNQDPWTF | 11 |
| 4C2 heavy chain | CARKVLFMRAMDYW | 14 |
| 4C2 kappa light chain | CQQSTEDPWTF | 11 |

**Table S2.** **Heavy- and light-chain complementarity-determining region 3 sequences.** Amino acid sequences and lengths of the heavy-chain CDR3 and κ light-chain CDR3 regions of antibodies 3A7, 3F4, 3D7, and 4C2.

| KL-BHA-3D7 complex | | | |
| --- | --- | --- | --- |
| HA: residue / atom | Fab : chain / residue / atom | Distance (Å) | Type of interaction |
| Ser39 / O | H / Trp107 / NE1 | 2.9 | H-bond |
| Thr37 / OG1 | H / Arg105 / NE | 2.9 | H-bond/salt bridge |
| Tyr40 / CD2 | H / Tyr103 / O | 3.0 | Aromatic vdW |
| Gly284 / O | H / Thr56 / OG1 | 3.1 | H-bond |
| Glu298 / O | H / Tyr59 / OH | 3.1 | H-bond |
| Val87 / N | H / Gly54 / O | 3.1 | H-bond |
| Ser285 / OG | H / Tyr50 / OH | 3.5 | H-bond |
| Glu235 / OE2 | H / Pro75 / CG | 3.5 | Van der Waals |
| NAG456 / O6 | H / Tyr103 / CE1 | 3.2 | Van der Waals |
| Glu182 / OE2 / chainB | H / Lys19 / NZ | 3.1 | Salt bridge |
| Asn303 / N | L / Tyr31 / OH | 3.2 | H-bond |
| Lys38 / NZ | L / Asp98 / OD1 | 3.2 | Salt bridge |
| KL-BHA- 4C2 complex | | | |
| HA: residue / atom | Fab : chain / residue / atom | Distance (Å) | Type of interaction |
| Lys298 / NZ | H / Asp58 / OD1 | 3.0 | Salt bridge |
| Arg38 / NH1 | H / Asp56 / OD1 | 3.0 | Salt bridge |
| Arg38 / O | H / Arg105 / NH2 | 3.1 | H-bond |
| Leu286 / O | H / Ser32 / OG | 3.1 | H-bond |
| Thr37 / OG1 | H / Arg105 / NH1 | 3.2 | H-bond |
| Ser285 / OG | H / Asp56 / OD2 | 3.3 | H-bond |
| Pro287 / CD | H / Ser32 / OG | 3.6 | Van der Waals |
| His40 / NE2 | H / Leu102 / CD2 | 3.6 | Van der Waals |
| Tyr299 / CD1 | H / Tyr60 / OH | 3.7 | Aromatic/H-bond |
| Asn303 / CB | L / Tyr32 / CE1 | 3.6 | Van der Waals |
| Ser305 / N | L / Tyr32 / OH | 3.3 | H-bond |
| Lys304 / O | L / Tyr32 / OH | 3.8 | H-bond (weak) |
| NAG460 / O6 | L / Asn34 / OD1 | 2.9 | H-bond |
| NAG459 O3 | L / Asn34 / ND2 | 3.4 | H-bond |
| KL-BHA-3F4 complex | | | |
| HA: residue / atom | Fab : chain / residue / atom | Distance (Å) | Type of interaction |
| Gln162 / OE1 | L / Asp28 / OD2 | 2.9 | H-bond |
| Asp163 / OD2 | L / Val29 / O | 2.9 | H-bond |
| Lys136 / NZ | L / Asp28 / O | 3.0 | Salt bridge |
| Lys240 / O | H / Tyr104 / N | 3.0 | H-bond |
| Gln199 / OE1 | H / Gln107 / NE2 | 3.1 | H-bond |
| Gly141 / O | H / Arg59 / NH2 | 3.1 | Salt bridge |
| Ser140 / OG | H / Asp105 / OD2 | 3.2 | H-bond |
| Gln241 / OE1 | H / Asp105 / OD2 | 3.3 | H-bond |
| Ile160 / CD1 | L / Tyr92 / OH | 3.4 | Van der Waals |
| Trp158 / CH2 | H / Tyr106 / OH | 3.4 | Van der Waals |
| Asn150 / OD1 | L / Ser94 / CB | 3.4 | Van der Waals |
| Ser242 / N | H / Tyr103 / CD1 | 3.5 | Van der Waals |
| Arg202 | L / Trp50 | 3.6 | Cation–π |
| Lys166 / NZ | L / Ser30 / OG | 3.7 | Van der Waals |
| Arg149 / NH2 | L / Asp1 / OD2 | 3.7 | Salt bridge |
| Leu239 / CD1 | H / Trp50 / CH2 | 3.7 | Van der Waals |
| Gly238 / O | H / Tyr52 / CE2 | 3.6 | Van der Waals |
| Leu203 / CD1 | H / Tyr106 / CE2 | 3.8 | Van der Waals |
| Phe95 / CE1 | H / Asp105 / OD2 | 4.0 | Van der Waals |

**Table S3.** **List of antibody:HA interactions detailing the residues involved and the distance and nature of the interaction.**
